# Supplementary material for: SARS-CoV-2 protein ORF8 limits expression levels of Spike antigen and facilitates immune evasion of infected host cells
Source: J Biol Chem. 2023 Jun 23;299(8):104955. doi: 10.1016/j.jbc.2023.104955 (PMC10289268; doi:10.1016/j.jbc.2023.104955)
Supplement: Supporting Table S1 — Specifications of anti-SARS-CoV-2 human sera. A. Source of COVID-19–negative or –positive (convalescent) sera. B. Source of COVID-19 vaccinated sera. three Pfizer and three Moderna, collected before first shot (pre-vaccination) and after second shot (post-vaccination). [file mmc1.docx]

**A.**

| COVID-19 | donor # | age | sex | ethnicity | identifier # |
| --- | --- | --- | --- | --- | --- |
| negative | 1 | 32 | male | Caucasian | V901A |
|  | 2 | 48 | male | Asian | V906A |
|  | 3 | 24 | female | Caucasian | V911A |
| positive (collected 06/17/20) | 1 | 26 | male | Caucasian | RPGG68 |
|  | 2 | 26 | female | Hispanic | TGRJLK |
|  | 3 | 26 | female | Hispanic | GT4DZ4 |

**B.**

| brand | donor # | age | sex | race | vaccination and identifier # | |
| --- | --- | --- | --- | --- | --- | --- |
|  |  |  |  |  | pre- | post- |
| Pfizer | 1 | 32 | male | Caucasian | V901A | V901C44 |
|  | 2 | 48 | male | Asian | V906A | V906C11 |
|  | 3 | 24 | female | Caucasian | V911A | V911C8 |
| Moderna | 1 | 34 | male | Caucasian | V902A | V902C22 |
|  | 2 | 47 | male | Caucasian | V903A | V903C20 |
|  | 3 | 46 | female | Asian | V909A | V909C22 |
