# Supplementary figures and images for: SARS-CoV-2 protein ORF8 limits expression levels of Spike antigen and facilitates immune evasion of infected host cells
Source: J Biol Chem. 2023 Jun 23;299(8):104955. doi: 10.1016/j.jbc.2023.104955 (PMC10289268; doi:10.1016/j.jbc.2023.104955)

**A**

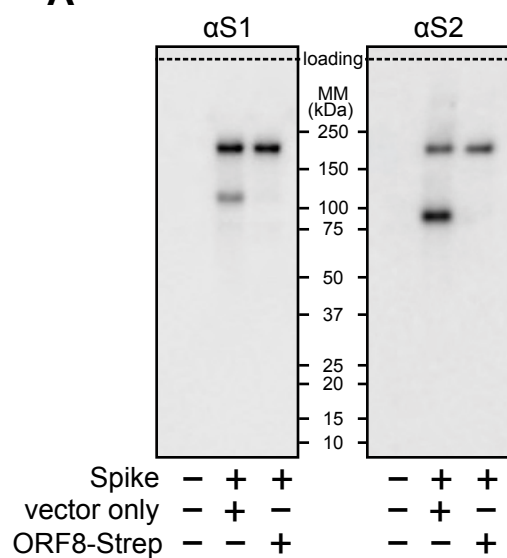

**B**

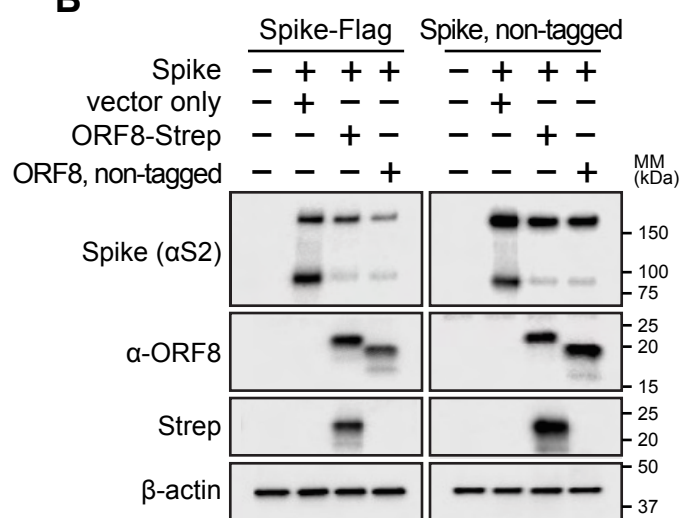

Supplement: Supporting Figure S1 — Validation of using C-terminal-tagged Spike or ORF8 constructs.A and B, HEK293T cells co-transfected with plasmids encoding Spike-Flag (A and B) or non-tagged Spike (B) or ORF8-Strep (A and B) or non-tagged ORF8 (B) were lysed and evaluated by immunoblot analysis using antibodies against S2 (detects uncleaved and S2 fragment of Spike) (A and B), S1 (detects uncleaved and S1 fragment of Spike), Strep (detects ORF8-Strep) (B), ORF8 (detects both ORF8-Strep and non-tagged ORF8) (B) and β-actin. The data represent three independent experiments. [file mmc2.pdf]

**A**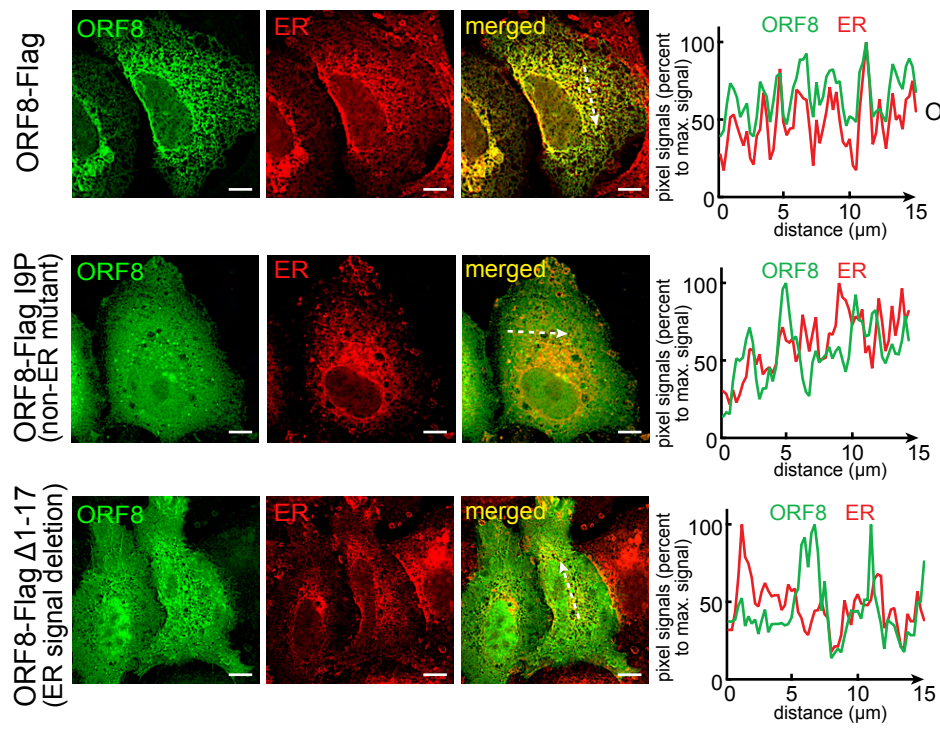**B**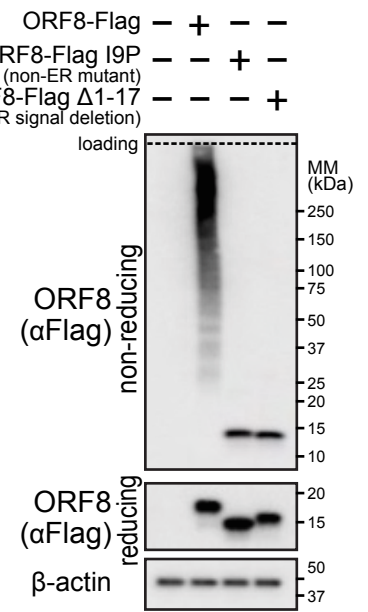

Supplement: Supporting Figure S2 — I9P mutation incapacitates ORF8 translocation to ER.A, A549 cells transfected with a plasmid encoding ORF8-Flag, ORF8-Flag I9P (non-ER mutant), or ORF8-Flag Δ1-17 (ER signal deletion) were fixed, permeabilized, and immunostained for PDI (ER marker) and Flag (ORF8), and analyzed by fluorescence confocal microscopy imaging. White scale bars = 10 μm. The pixel intensities of ORF8 and PDI along the dashed arrow are plotted. B, HEK293T cells transfected with a plasmid encoding ORF8-Flag, ORF8-Flag I9P, or ORF8-Flag Δ1-17 were lysed and evaluated by immunoblot analysis using antibodies against Flag (ORF8) or β-actin under reducing or non-reducing (protein interactions through disulfide bonds are preserved) conditions. The data represent three independent experiments. [file mmc3.pdf]

**A**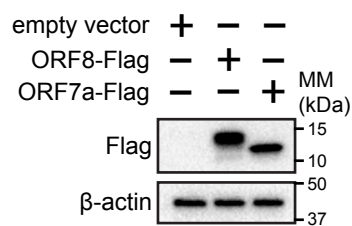**B**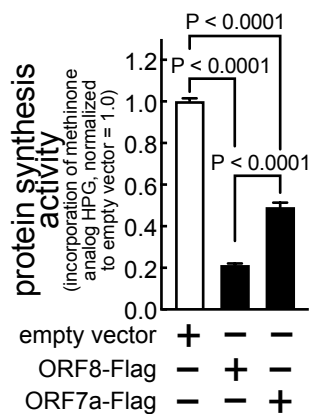**C**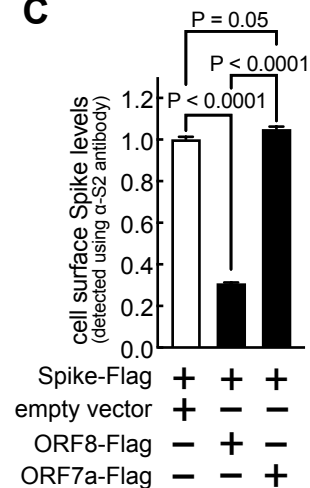**D**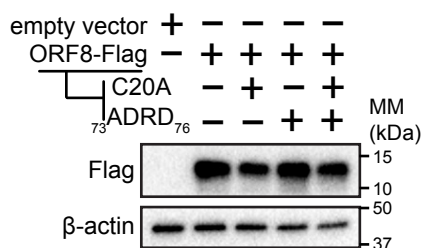**E**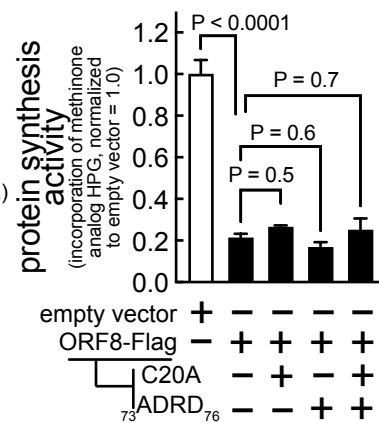**F**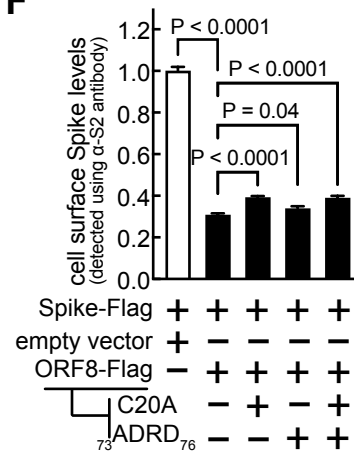

Supplement: Supporting Figure S3 — Effect of ectopic expression of ORF7a or ORF8 mutants on protein synthesis and cell surface Spike levels.A–F, HEK293T cells were transfected with a plasmid encoding ORF7a-Flag (A–C), ORF8-Flag (A–F), or modified ORF8-Flag (C20A (Cys20 replaced with Ala), 73ADRD76 (73YIDI76 replaced with 73ADRD76) (D–F). After 30 min incubation in the absence (A, C, D and F) or presence (B and E) of HPG (methionine analog that can be fluorescently labeled), the cells were lysed for immunoblot analysis for expression levels of the Flag tagged constructs (A and D), or fixed, permeabilized, fluorescently labeled and analyzed by flow cytometry for incorporated HPG (B and E), or immunostained with antibodies against Spike S2 and analyzed by flow cytometry for cell surface Spike levels (C and F). The data are representative of or combined from three independent experiments, and are presented as mean ± s.d. Statistical significance was analyzed using one-way ANOVA (Tukey’s test). [file mmc4.pdf]
